# Breakfast at Tiffany’s: encouraging all the best and brightest diamonds into cardiothoracic surgery

**DOI:** 10.1093/icvts/ivac249

**Published:** 2022-10-26

**Authors:** Miia Lehtinen, Maroua Eid

**Affiliations:** Department of Cardiac Surgery, Heart and Lung Center, Helsinki University Hospital and University of Helsinki, Helsinki, Finland; Department of Cardiac Surgery, University Hospital of Angers and University of Angers, Angers, France

**Keywords:** Residency, Training, Female resident

An informal breakfast at the 24th annual meeting of the Society of Thoracic Surgeons initiated a series of events that have affected the profile of our cardiothoracic community ever since [1]. After that breakfast, the association of Women in Thoracic Surgery was founded in 1986 with the mission to mentor and enhance opportunities for women thoracic surgeons in the USA. In 2020, >30 years after that famous breakfast in the USA, those waves finally hit the shores on the other side of the Atlantic: The Women in Cardio-Thoracic Surgery Committee was established under the European Association for Cardio-Thoracic Surgery. And in 2021, under the leadership of Prof. Jolanda Kluin, the committee organized its first annual meeting session in Barcelona.

‘I have never tried that before so I should definitely be able to do that’.—Pippi Longstocking

Addressing the issue of disparities between men and women can seem redundant, since this dichotomy is well recognized in the professional world. It is nevertheless essential in our surgical field since it is still a male-dominated world: most recent data from the public report of the American Association of Medical Colleges (AAMC) in 2019 on active physicians sex and specialtyindicates that 92% of practicing cardio-thoracic surgeons are men [2]. Therefore, the surgical work environment is designed for this majority, with all its equipment largely tailored according to the size, strength and reasoning of male surgeons [3]. Despite these obstacles, female surgeons have excelled in their surgical domains, as emphasized by high-impact journals: in the *British Medical Journal*, Wallis *et al.* [4] reported that patients operated by female surgeons were not getting worse treatment but, on the contrary, were less likely to die within 30 days. In the *Annals of Thoracic Surgery*, Rong *et al.* [5] reported their results regarding transcatheter aortic valve replacement performed by female operators and found the risk of adverse events to be non-inferior to male operators.

Sadly, despite this documented equality in operative results, the academic career ladder still seems steeper for female surgeons: women with an MD are less likely to author a paper than men with the same degree and instead, a PhD becomes much more essential for women [6]. They also occupy fewer higher positions in the academic field, as reported by the last survey on gender bias in Europe: men have an associate professor position almost twice as often as do women surgeons and a professor position almost four times as often (6% vs 11% and 6% vs 22%, respectively) [7]. The steeper ladder affects particularly the cardiothoracic domain, where female and ethnic minorities are underrepresented [8]. Moreover, women get less podium time in meetings [9]. This lack of inclusivity seems to exist already in the grass root level of academia since despite half of the medical students being female, only 24% of cardiothoracic residents are women [10]. Factors contributing to obstacles in the education pipeline of potential women surgeons are, e.g. concerns related work-life balance and lack of especially same-sex mentors [10].

‘I do not wish to give (women) a first place, still less a second one—but the complete freedom to take their true place, whatever it may be’.—Elizabeth Blackwell

The Women in Cardio-Thoracic Surgery Committee currently consists of seven female surgeons from various career levels and countries: Prof. Jolanda Kluin (The Netherlands); Dr Indu Deglurkar (UK); Dr Lena Emrich (Germany); Dr Francesca D’Auria (Italy); Dr Julie Cleuziou (Germany); Dr Miia Lehtinen (Finland); and Dr Maroua Eid (France). This heterogeneity seems essential, allowing all female surgeons to relate to the committee.

The committee holds an important mission: establishing a network for *female surgeons* [11]. Such connections will boost female surgeons’ confidence, showing them that targeting your goals can bear fruit, while retaining promising residents in the field. Upcoming projects articulate around the creation of an effective mentorship program and supporting young female surgeons with a new travel fellowship. In an effort to increase female surgeons’ visibility and to offer a dedicated space for idea exchange, the committee is developing a webinar series, addressing universal issues of inclusivity as well as means for improvement.

Gender should not intervene in one’s career choices; only abilities and perseverance should be considered. A shift towards an increased representation of female surgeons is to be expected in our field, and as suggested by Shemanski *et al.* ‘attention should be given to the potential unconscious bias in leadership in cardiothoracic surgery’ [9]. For our primary goal should be clear: ensuring that our patients will be treated by the best surgeons possible, regardless of surgeon gender.
